# Drug-Induced Fatty Liver Disease (DIFLD): A Comprehensive Analysis of Clinical, Biochemical, and Histopathological Data for Mechanisms Identification and Consistency with Current Adverse Outcome Pathways

**DOI:** 10.3390/ijms25105203

**Published:** 2024-05-10

**Authors:** Ernesto López-Pascual, Ivan Rienda, Judith Perez-Rojas, Anna Rapisarda, Guillem Garcia-Llorens, Ramiro Jover, José V. Castell

**Affiliations:** 1 Department of Biochemistry and Molecular Biology, University of Valencia, 46010 Valencia, Spain; 2 Joint Research Unit in Experimental Hepatology, Health Research Institute La Fe, 46026 Valencia, Spain; 3 Pathology Department, Hospital Universitario y Politécnico La Fe, 46026 Valencia, Spain; 4 Centro de Investigación Biomédica en Red de Enfermedades Hepáticas y Digestivas (CIBERehd), Instituto de Salud Carlos III, 28029 Madrid, Spain

**Keywords:** drug-induced fatty liver disease, liver steatosis, steatohepatitis, adverse outcome pathways, mitochondrial damage

## Abstract

Drug induced fatty liver disease (DIFLD) is a form of drug-induced liver injury (DILI), which can also be included in the more general metabolic dysfunction-associated steatotic liver disease (MASLD), which specifically refers to the accumulation of fat in the liver unrelated to alcohol intake. A bi-directional relationship between DILI and MASLD is likely to exist: while certain drugs can cause MASLD by acting as pro-steatogenic factors, MASLD may make hepatocytes more vulnerable to drugs. Having a pre-existing MASLD significantly heightens the likelihood of experiencing DILI from certain medications. Thus, the prevalence of steatosis within DILI may be biased by pre-existing MASLD, and it can be concluded that the genuine true incidence of DIFLD in the general population remains unknown. In certain individuals, drug-induced steatosis is often accompanied by concomitant injury mechanisms such as oxidative stress, cell death, and inflammation, which leads to the development of drug-induced steatohepatitis (DISH). DISH is much more severe from the clinical point of view, has worse prognosis and outcome, and resembles MASH (metabolic-associated steatohepatitis), as it is associated with inflammation and sometimes with fibrosis. A literature review of clinical case reports allowed us to examine and evaluate the clinical features of DIFLD and their association with specific drugs, enabling us to propose a classification of DIFLD drugs based on clinical outcomes and pathological severity: Group 1, drugs with low intrinsic toxicity (e.g., ibuprofen, naproxen, acetaminophen, irinotecan, methotrexate, and tamoxifen), but expected to promote/aggravate steatosis in patients with pre-existing MASLD; Group 2, drugs associated with steatosis and only occasionally with steatohepatitis (e.g., amiodarone, valproic acid, and tetracycline); and Group 3, drugs with a great tendency to transit to steatohepatitis and further to fibrosis. Different mechanisms may be in play when identifying drug mode of action: (1) inhibition of mitochondrial fatty acid β-oxidation; (2) inhibition of fatty acid transport across mitochondrial membranes; (3) increased de novo lipid synthesis; (4) reduction in lipid export by the inhibition of microsomal triglyceride transfer protein; (5) induction of mitochondrial permeability transition pore opening; (6) dissipation of the mitochondrial transmembrane potential; (7) impairment of the mitochondrial respiratory chain/oxidative phosphorylation; (8) mitochondrial DNA damage, degradation and depletion; and (9) nuclear receptors (NRs)/transcriptomic alterations. Currently, the majority of, if not all, adverse outcome pathways (AOPs) for steatosis in AOP-Wiki highlight the interaction with NRs or transcription factors as the key molecular initiating event (MIE). This perspective suggests that chemical-induced steatosis typically results from the interplay between a chemical and a NR or transcription factors, implying that this interaction represents the primary and pivotal MIE. However, upon conducting this exhaustive literature review, it became evident that the current AOPs tend to overly emphasize this interaction as the sole MIE. Some studies indeed support the involvement of NRs in steatosis, but others demonstrate that such NR interactions alone do not necessarily lead to steatosis. This view, ignoring other mitochondrial-related injury mechanisms, falls short in encapsulating the intricate biological mechanisms involved in chemically induced liver steatosis, necessitating their consideration as part of the AOP’s map road as well.

## 1. Introduction

Metabolic dysfunction-associated steatotic liver disease (MASLD) is a pathological condition associated with the accumulation of fat in liver cells unrelated to alcohol, leading initially to hepatic malfunction and then to inflammation and scarring. MASLD encompass histopathological manifestations such as macrovesicular and microvesicular steatosis, steatohepatitis, and fibrosis [1,2]. Its high prevalence is increasing, which represents a health problem in Western countries.

Apart from a metabolic ontogeny, drugs have also been associated with steatotic liver disease (SLD) causing per se liver malfunction or exacerbating pre-existing fatty liver or steatohepatitis, and in some cases promoting the transition of fatty liver to fibrosis or cirrhosis. Drug-induced fatty liver disease (DIFLD) is a significant contributor to overall drug-induced liver injury (DILI), a disease that increases with age, multi-drug usage, and preexisting liver pathologies. The pathogenesis of DIFLD involves different mechanisms, among them mitochondrial dysfunction, impaired ATP production, and fatty acid oxidation. Drugs like steroid hormones can exacerbate the pathogenetic mechanisms leading to steatohepatitis, while other drugs like tamoxifen and irinotecan have been shown to precipitate latent fatty liver disease.

In the present review, our focus has been centred on examining the clinical features of DIFLD patients and the mode of action of causal drugs extensively documented in the literature. We also sought to categorize DIFLD drugs based on their clinical and pathological outcomes. This is intended to facilitate the identification of additional molecular initiating events (MIEs), thus serving as a wellspring of novel concepts to refine existing adverse outcome pathways (AOPs).

To facilitate reader comprehension, this review adopts a structured approach. First, it delineates the relationship between MASLD and DIFLD. Subsequently, it delves into the histological aspects of DIFLD, surveying the characteristic liver injury patterns such as macrovesicular and microvesicular steatosis, steatohepatitis, and fibrosis. Following this, it thoroughly examines the clinical manifestations of DIFLD, encompassing symptoms, biochemical changes, and contemporary diagnostic modalities such as liver biopsy, nuclear magnetic resonance, and ultrasonography. The subsequent section undertakes the classification of medications inducing DIFLD based on clinical and pathological criteria. It then compiles a detailed exploration of the mechanisms underpinning drug-induced steatosis and liver damage, including mitochondrial dysfunction, lipid metabolism aberrations, and alternative pathways like increased de novo lipogenesis (DNL) and compromised lipid export mechanisms via very low-density lipoprotein (VLDL), along with the involvement of nuclear receptors (NRs) and transcription factors (TF). Additional sections endeavor to categorize steatosis-inducing drugs by their mechanisms and clinical outcomes, drawing from existing literature. Last, even though recent advancements have significantly contributed to enhance our understanding DIFLD by the development of AOPs (https://aopwiki.org/, accessed on 15 February 2024), the paper concludes by discussing current AOPs and advocating for the integration of supplementary factors to achieve a holistic comprehension of DIFLD.

## 2. Metabolic Dysfunction-Associated Steatotic Liver Disease (MASLD)

Liver steatosis, or SLD, is characterised by the buildup of excess fat in the liver, manifesting as an abnormal accumulation of lipids within hepatocytes. In the past, excessive alcohol consumption was the principal cause of liver steatosis in humans. Nowadays, other causes of steatosis are more prevalent and, hence, the newly defined MASLD specifically refers to the accumulation of fat in the liver, unrelated to alcohol intake [2].

MASLD covers a wide range of pathological conditions, ranging from simple accumulation of fat in the liver (steatosis) to more complex states involving inflammation and damage to liver cells (metabolic dysfunction-associated steatohepatitis, MASH). Additionally, MASLD is linked to several other health issues, including obesity, insulin resistance, elevated blood sugar, high serum lipid levels, hypertension, and cardiometabolic risk factors. The prevalence of this condition is increasing, particularly in Western countries, largely due to lifestyle factors such as unhealthy dietary choices and sedentary habits.

Under the umbrella term of SLD, several subtypes exist. These include alcohol-associated liver disease, the already mentioned MASLD, and mixed phenotypes (metabolic alcohol-related liver disease, MetALD), which collectively encompass most patients with fatty liver. However, it is important to recognize that drugs and other chemicals can also trigger SLD. When this occurs, it is referred as DIFLD. Surprisingly, up to 2% of cases initially diagnosed as MASLD are actually caused by drugs. Unfortunately, distinguishing between these two conditions relies solely on proper identification of exposure to a steatogenic compound [1].

**Figure 1:**
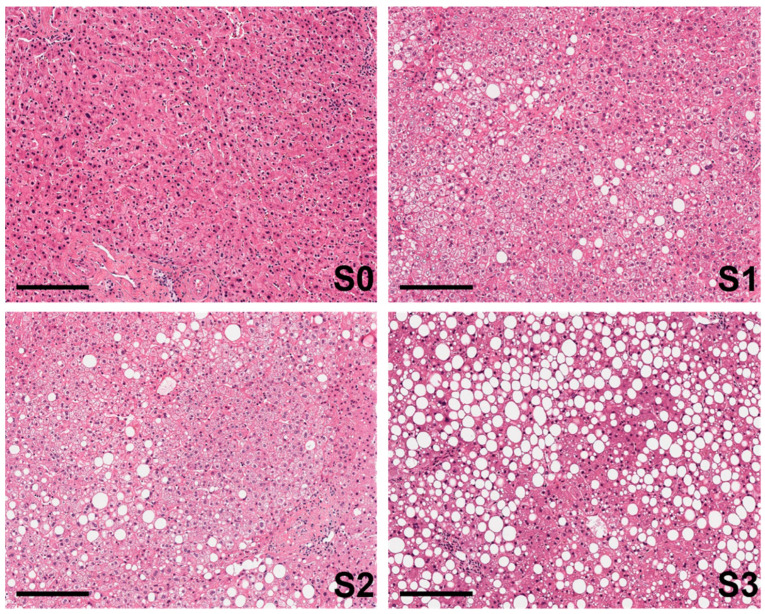
Scoring of the degree of steatosis in human liver biopsies. Steatosis refers to the abnormal accumulation of lipids in hepatocytes after liver biopsy staining with haematoxylin–eosin (H&E). The grading of steatosis typically follows a scale from (**S0**–**S3**), indicating the severity of the condition. Micrographs show (10× magnification), representative images, black bar equals 200 μm.

Historically, the diagnosis and grading of steatosis have relied on examining liver biopsies. Key evaluation parameters have included the size of lipid droplets, the percentage of cytoplasmic volume occupied by lipids, and the extent of affected parenchymal area [3,4]. More recent classifications make use of a semiquantitative scale ranging from S0 to S3, correlating with the degree and severity of the disease. S0 denotes no steatosis, with the liver exhibiting a normal appearance and lacking any notable accumulation of fat within hepatocytes. S1 indicates mild steatosis, i.e., a small amount of fat accumulation within the hepatocytes with 5–33% being affected. S2 stage indicates moderate steatosis, with a moderate amount of fat accumulation, affecting typically between 34–66% of hepatocytes. S3 indicates severe steatosis, with a significant amount of fat accumulation within the hepatocytes, and more than 66% of them affected [5,6,7] (Figure 1). 

## 3. Drug-Induced Fatty Liver Disease (DIFLD)

DIFLD can be considered as a part of the much broader DILI phenomenon. The estimated annual incidences of DILI vary in different population-based studies and range from 2.7 to 19.1 cases per 100,000 inhabitants and among them, approximately 27% display some form of steatosis in the histological injury examination [8,9]. A bi-directional relationship between DILI and MASLD is likely to exist: while certain drugs can cause MASLD by acting as pro-steatogenic factors, MASLD may make hepatocytes more vulnerable to drugs [10]. Having a pre-existing MASLD significantly heightens the likelihood of experiencing DILI from certain medications. Therefore, in obese people who manage multiple health conditions through polypharmacy, the different drugs could significantly contribute to the risk of steatosis and liver injury. Despite steatosis being a well-characterised feature and a monitored histopathological finding for diagnosis, it lacks specificity. Thus, the prevalence of steatosis within DILI may be biased by pre-existing MASLD, and it can be concluded that the genuine true incidence of DIFLD in the general population remains unknown.

The histological appearance of the fat deposition varies. In macrovesicular steatosis, accumulation of large single lipid droplets is observed. These lipid droplets displace the cell nucleus towards the periphery and is a predominant feature of DIFLD. Frequently it is accompanied by lobular inflammation (typically mild and mixed, including polymorphonuclear leukocytes and mononuclear cells). Most apparent near steatotic liver cells are often observed in zone 3. Occasionally, perisinusoidal fibrosis is seen in zone 3 [11].

**Figure 2:**
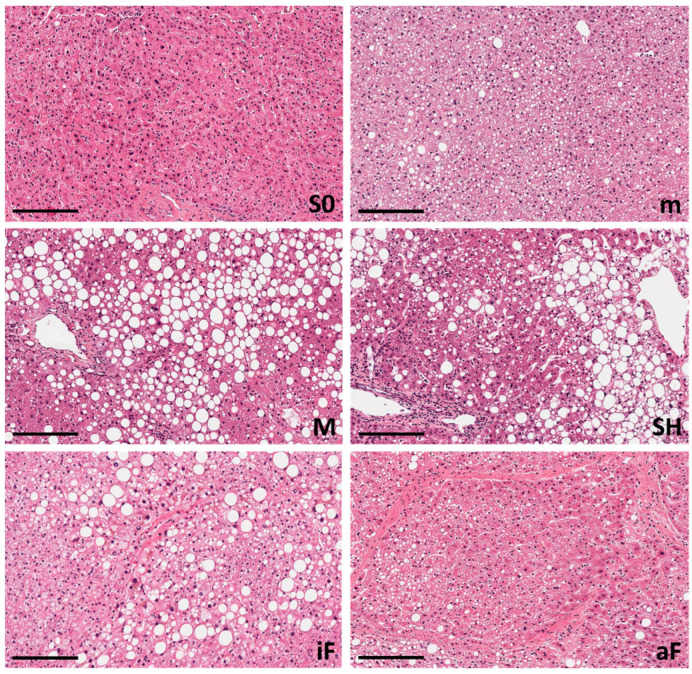
Pathological changes in liver tissue in DIFLD. Micrographs were obtained from liver biopsies of DIFLD patients, after processing and H&E staining. Several typical representative patterns of the disease are shown; black bar equals 200 μm: unaffected ((**S0**), 10× magnification), microsteatosis ((**m**), 10×), macrosteatosis ((**M**), 10×), steatohepatitis ((**SH**), 20×), incipient fibrosis ((**iF**), 20×) and advanced fibrosis ((**aF**), 10×).

In contrast, microvesicular steatosis, although less common, is more severe, involving the accumulation of small fat droplets within liver cells. The appearance of hepatocytes is distended with foamy cytoplasm containing numerous small lipid vesicles (usually less than 1 μm in diameter), within cytoplasm (Figure 2).

It may coexist with other features like hepatocellular ballooning, portal fibrosis, lobular inflammation, and portal inflammation. Steatohepatitis denotes an inflammatory status of liver cells that may evolve into fibrosis of liver tissue [12].

Drugs that promote or mimic MASLD pathogenic factors, such as insulin resistance and/or imbalances in lipid homeostasis, are believed to primarily induce macrovesicular steatosis. This accumulation of neutral fats (mainly triglycerides) more likely results from an imbalance in the liver’s uptake and processing of fatty acids from circulation, as well as in de novo synthesis and exportation. When the net balance surpasses the liver’s processing capacity, intracellular lipids buildup in large vesicles. Conversely, microvesicular steatosis is mainly linked to acute mitochondrial dysfunction [13]. Another form of fat accumulation, phospholipidosis, is associated with defective lipophagy.

In certain individuals, drug-induced steatosis may be accompanied by concomitant injury mechanisms such as oxidative stress, cell death and inflammation, which leads to the development of drug-induced steatohepatitis (DISH) [14]. DISH bears similarities to MASH, marked by inflammation and occasionally fibrosis. However, it tends to be notably more severe clinically, with poorer outcomes and prognosis. (Figure 2).

Mitochondrial damage is central in DIFLD, and it is also frequently involved in the onset of DISH. An intriguing scenario is the one provided by drugs inducing steatosis in the absence of severe mitochondrial damage. Here, the involvement of mild-to-moderate inhibition of mitochondrial fatty acid oxidation, together with increased DNL, and/or impairment lipid export via VLDL all seem to play a key role [10,15,16].

## 4. Clinical Features in DIFLD

Clinical symptoms and biochemical alterations in DIFLD are usually minimal or moderate in the early stages of disease. The clinical symptoms of DIFLD are not specific to this pathology and are rather shared with other liver affections. They can vary depending on the severity of the condition and, eventually, the specific drug involved (Table 1). Common and non-specific clinical symptoms include fatigue, abdominal discomfort or pain, jaundice, weight loss, enlarged liver, and abdomen or leg swelling by fluid retention. Quite frequently, individuals with DIFLD may not experience any symptoms initially, and the condition may only be detected through medical imaging analyses. 

Besides clinical symptoms, DIFLD may also coincide with various analytical signs, detectable through laboratory tests, aiding in the diagnosis and monitoring of the condition. e.g., elevated alanine aminotransferase (ALT) and aspartate aminotransferase, in the liver inflammation stage, alkaline phosphatase (ALP) and gamma-glutamyl transferase if cholestasis is also present. Elevated serum cholesterol and triglycerides may be observed due to impaired lipid metabolism associated with liver steatosis. Decreased levels of albumin and total protein may also occur, indicating an impaired liver function. Coagulation profile, with prolonged prothrombin time or an elevated international normalized ratio suggest impaired synthesis of clotting factors by a dysfunctional liver as well. The onset and latency period of DIFLD before clinical symptoms emerge can vary widely from weeks to months after drug exposure [17].

**Figure 3:**
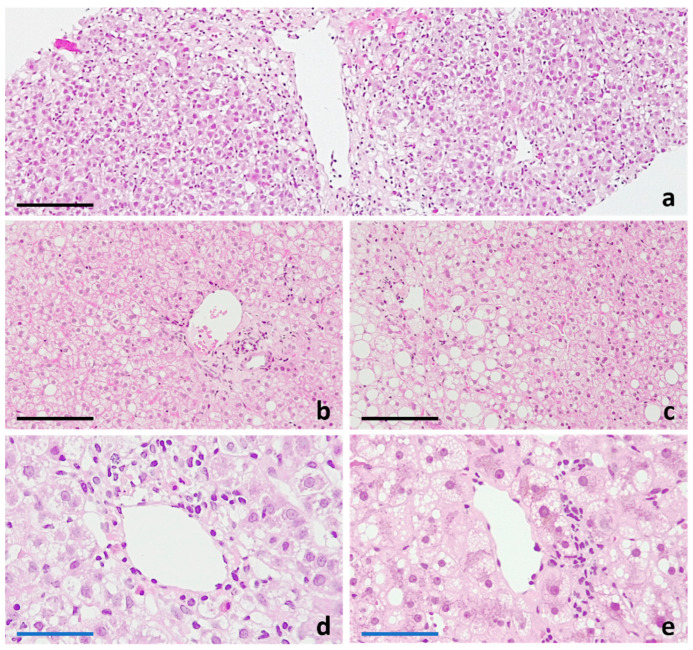
Steatotic pattern in toxic hepatic injury. Steatosis and steatohepatitis can be found in combination with other patterns of hepatic injury, like acute coagulative necrosis ((**a**), 10× magnification). While periportal parenchymal areas are typically respected ((**b**), 20×), centroacinar (perivenular) zones are typically affected by steatotic and steatohepatitic lesions ((**c**), 20×; (**d**), 40× and (**e**), 40×). Both, predominantly macrovesicular ((**c**), 20×) or microvesicular steatosis ((**e**), 40×) can constitute the major finding in this pattern of lesion. The histological findings of DIFLD are indistinguishable from those of other causes of steatosis. Black bar equals 200 μm. Blue bars equals 75 μm.

Regarding diagnosis by medical imaging, nuclear magnetic resonance and ultrasonography have gained relevance for a non-invasive, quantitative estimation of a patient’s liver steatosis. However, for precise differential DIFLD/DISH diagnosis, the liver needle biopsy remains the gold standard, as it also allows us to determine the extent of cell and tissue damage, liver inflammation and fibrosis [16,18]. Thus, in some cases, confirmatory diagnosis of steatosis and of the degree of severity is made by histological analysis. The most obvious finding in the histological examination are the lipid droplets. However, DIFLD may have different histochemical patterns (Figure 2). The distinction between macrovesicular and microvesicular steatosis lies in the morphology and arrangement of lipid vesicles in hepatocytes (Figure 3). Macrovesicular steatosis shows few big lipid vacuoles per cell, while microvesicular steatosis presents with numerous small cytoplasmic lipid droplets. In both instances the disease image may display inflammatory features when DISH develops.

In macrovesicular steatosis, large lipid vesicles, mostly containing triglycerides, frequently invade a large region of the cytoplasm and are visibly larger than the nucleus. Hepatocytes are usually enlarged and distended, displacing the nucleus and other cellular organelles towards the cell periphery [14]. In addition, the normal cell architecture is disrupted, impacting liver’s functionality.

Hepatocyte ‘ballooning’ may also be observed in DIFLD. This is a widely used term in liver histopathology that indicates hepatocyte degeneration associated with enlargement, swelling, rounding and characteristic reticulated cytoplasm. These cells have a diameter about 1.5–2 times greater than that of normal hepatocytes [19]. Hepatocytic ballooning is linked to the buildup of lipid droplets, expansion of the endoplasmic reticulum, and damage to intermediate cytoskeletal filaments. The microtubule cytoskeleton, which is essential for normal efficient vesicle transport in the hepatocyte, is destroyed [20], causing nascent protein retention and an increase in the diameter of hepatocytes. Furthermore, higher risk of developing liver-related complications and greater severity of liver disease has been associated with hepatocyte ballooning.

Sometimes, drug-induced macrovesicular steatosis (Figure 3) may exhibit an uneven zonal distribution within the liver lobules. This means that the accumulation of fat is more prominent in certain areas or zones of the liver than in others. It is essential to consider this aspect when acquiring and scrutinising a liver needle biopsy for diagnostic purposes. Macrovesicular steatosis, when mild, may be clinically silent and can be reversible [16,21].

Drug-induced microvesicular steatosis is very frequently associated with mitochondrial damage [13]. In certain cases, steatosis develops with a preliminary formation of small cytoplasmic lipid droplets that further coalesce to generate large vesicles (macrovesicular steatosis). However, in microvesicular steatosis, usually associated with liver failure and more profound hypoglycemia and acidemia, the hepatocyte cytoplasm is filled with numerous stable small lipid vesicles and the nucleus remains in the centre of the cell [22,23]. The severe impairment of mitochondrial fatty acid oxidation leads to increased esterification of fatty acids into triglycerides, which are also the primary lipids in microvesicular steatosis. In macrovesicular steatosis, mixed large and small droplets can also be observed [14,22].

Depending on the distinct pathogenic mechanism involved for a given drug, simple steatosis or the more severe steatohepatitis (DISH) may develop, with many cases initially displaying acute microvesicular injury. Steatohepatitis, a more serious liver injury status, is histologically characterised by lobular inflammation, ballooning degeneration, hyaline Mallory–Denk bodies, and, sometimes, perisinusoidal fibrosis [24]. Steatohepatitis frequently evolves towards fibrosis and cirrhosis and ultimately becomes the trigger of a primary hepatocarcinoma, all of them with deleterious consequences for the individual, deeply affecting liver function and health.

Regarding causal DIFLD drugs, some of them have been associated with the causation of weakness, fever, fatigue, nausea, and abdominal pain, whereas others only cause jaundice or no symptoms at all (Table 1). Regarding laboratory tests, they typically show minimal or modest elevations of aminotransferases and ALP [25]. Certain drugs are also associated with lactic acidosis (Table 1).

## 5. Drugs Causing DIFLD and Their Classification

There are a variety of medications that can cause DIFLD. For instance, some steroids, antiretrovirals, chemotherapy drugs, and antibiotics have been frequently found to trigger fatty liver as a side effect. Either these drugs per se, or their metabolites generated by cytochrome P450, are ultimately responsible for disrupting lipid homeostasis, leading to an accumulation of fat within liver cells.

As anticipated above, the relationship between DILI and MASLD may be reciprocal: medications can cause MASLD by acting as pro-steatogenic factors, and pre-existing MASLD could be a predisposing condition for certain medications to more easily cause DILI [10]. Hence, clinicians should remain alert to the potential of drugs that trigger hepatic steatosis, and patients who are prescribed DIFLD-associated medications need to be monitored to detect possible hepatic side effects, particularly steatosis [26,27].

A literature review of clinical case reports allowed us to examine and evaluate the clinical features of DIFLD and their association with given drugs, enabling us to propose a classification of DIFLD drugs based on clinical outcomes and pathological severity characteristics. Three groups were found (Table 2): (1) drugs with low intrinsic toxicity (e.g., ibuprofen, naproxen, acetaminophen, irinotecan, methotrexate, and tamoxifen), but expected to promote/aggravate steatosis in patients with pre-existing MASLD or related conditions, e.g., obesity, metabolic syndrome, cardiometabolic risk factors; (2) drugs associated with steatosis and only occasionally with steatohepatitis (e.g., amiodarone, valproic acid, and tetracycline); and (3) drugs with a great tendency to transit to steatohepatitis and further to fibrosis accompanied by cholestasis (e.g., zidovudine, stavudine, and didanosine) (Table 2).

Considering that MASLD is emerging as a prevalent cause of liver disorders, impacting around 30% of the global adult population and posing a significant burden on health systems, and that it aligns with ongoing epidemics of obesity, type 2 diabetes, and metabolic syndrome, drugs falling under GROUP 1 may attain increased significance, and deserve more attention in the future [28].

Thus, one of the most frequently used painkillers, acetaminophen, which is safe when used at clinical dosages, may be deleterious for patients with pre-existing steatosis. Indeed, several studies have described the occurrence of susceptible populations (e.g., obese and MASLD) in which acetaminophen worsened the baseline liver steatosis and injury [29,30,31].

Similarly, widely used nonsteroidal anti-inflammatory drugs (NSAIDs), such as ibuprofen and naproxen, have also been reported to induce hepatic steatosis (usually microvesicular) by inhibiting β-oxidation of short- and medium-chain fatty acids [26]. In certain situations, NSAIDs can promote steatohepatitis and the progression of MASLD in patients with predisposing factors such as obesity and metabolic syndrome. Also, the impairment of the intestinal barrier by NSAIDs can lead to bacterial translocation and toxic derivatives that reach the liver via the portal circulation, promoting an endotoxemia that could lead to liver inflammation and MASH development [32]. In contrast, a retrospective study found that non-aspirin NSAIDs were associated with lowering fibrosis scores in patients with MASLD, suggesting that NSAID use, by blocking inflammatory reactions, might be associated with a lower risk for fibrosis development in these patients [33]. Hence, the impact of NSAIDs on the occurrence of steatohepatitis may vary depending on factors such as the specific NSAID used, the individual’s health status, and the presence of additional risk factors.

Tamoxifen, methotrexate, and irinotecan have been associated with worsening steatohepatitis/steatosis in the presence of preexisting cardiometabolic risk factors, particularly obesity. In this way they can contribute to the progression of pre-existing steatosis towards MASH, fibrosis or cirrhosis [27,34].

GROUP 2 comprises drugs that tend to cause liver steatosis. The type of fat accumulation differs among causing drugs and is related to the type of perturbation they cause in hepatocytes. Macrovesicular steatosis is the predominant pattern in MASLD, and some drugs share common pathogenic factors such as insulin resistance and imbalance between fat gain and loss. 

Microvesicular steatosis is a potentially more severe liver injury and is linked to more acute DILI episodes [19]. It is associated with more specific disorders in hepatocyte metabolism. For instance, certain drugs and toxins can disrupt fatty acid β-oxidation (FAO) or the synthesis and secretion of lipoproteins, leading to the formation of smaller vesicles rather than large lipid droplets [35]. Drugs that can cause microvesicular steatosis include valproic acid, tetracycline, aspirin, ibuprofen, nucleoside reverse transcriptase inhibitors (NRTIs) such as didanosine, fialuridine, stavudine and zidovudine, as well as glucocorticoids (Table 3). Notwithstanding, genetic differences can also predispose an individual to develop microvesicular steatosis [35].

The development of macrovesicular or microvesicular steatosis by drugs can be influenced by a combination of factors affecting fatty acid uptake, metabolism, and processing, as well as specific conditions present in an individual, such as metabolic disorders, acute or chronic diseases, and genetic factors [35,36].

Regarding the drugs that cause steatohepatitis (GROUP 3), it is known that a triggering factor is prolonged therapy (more than 6 months). Moreover, differences in the metabolism and accumulation of the drug may also influence (as in the case of perhexiline maleate, as a consequence of CYP2D6 polymorphisms) [37].

In the case of NRTIs, studies have shown a natural history association of steatosis continued by steatohepatitis. The use of NRTIs, particularly dideoxynucleoside analogues such as didanosine and stavudine, caused hepatic steatosis in HIV-seropositive patients [38]. Additionally, thymidine analogues, particularly stavudine, have been linked to lipoatrophy, insulin resistance, and hyperlipidemia, which may also contribute to the development of steatosis [39].

The mechanism of NRTI-induced steatosis is not fully understood, but mitochondrial toxicity is believed to be responsible for their harmful effects [40]. Severe damage to mitochondrial DNA (mtDNA) by NRTIs, usually evolves towards an inflammatory scenario, paving the way towards steatohepatitis. Metabolic abnormalities are extremely common in HIV-infected persons on NRTIs. These metabolic abnormalities have also been associated with the development of MASH in HIV-infected patients [38].

Drug-induced steatohepatitis shares many pathological and clinical features with alcoholic steatohepatitis and MASH. The progression of DIFLD towards DISH frequently involves the production of damaging reactive oxygen species (ROS), which are responsible for oxidative stress and lipid peroxidation. These deleterious events subsequently trigger the production of different inflammatory cytokines such as TNFα and TGFβ that favour necroinflammation and fibrosis. Although the mitochondria produce the majority of ROS through mitochondrial respiratory chain (MRC) uncoupling, peroxisomal and microsomal (cytochrome P450) oxidations can also contribute. Nevertheless, over the past decades, several GROUP 2 drugs have also been shown to potentially induce steatohepatitis [37]. They include amiodarone [41], 5-Fluorouracil, irinotecan, methotrexate, perhexiline, tamoxifen and 4,4′-diethylaminoethoxyhexestrol [26,37,42,43], all of them have been found to induce a histologic picture of MASH in certain, but not all patients. In this context, advanced stage inflammation and fibrosis can be of variable severity and cirrhosis has also been occasionally reported for drugs such as amiodarone, perhexiline, and didanosine [13,37].

To delve into the associations between DIFLD drugs and the most important histopathological features, we reviewed the literature and searched information on four distinctive features: macrovesicular steatosis, microvesicular steatosis, steatohepatitis, and fibrosis. The number of articles supporting each feature was used as a relative incidence score. The association with fibrosis was extracted from LiverTox (Table 3). Although this revision was limited in its scope, some important differences among drugs could be observed: valproate, tetracycline, NRTIs, and NSAIDs are much more frequently associated with microvesicular than with macrovesicular steatosis. Conversely, antiarrhythmics (amiodarone and perhexiline) and chemotherapeutics are more frequently associated with macrovesicular steatosis as well as with steatohepatitis. Fibrosis, which usually develops after steatohepatitis, was reported in studies with amiodarone, some chemotherapeutics, and NRTIs.

Based on the most severe clinical outcomes (steatohepatitis and fibrosis), it is tempting to suggest that the DIFLD drugs of most concern are antiarrhythmics, chemotherapeutics, and NRTIs (Table 3).

## 6. Molecular Mechanisms Involved in the Onset of Drug-Induced Steatosis

**Figure 4:**
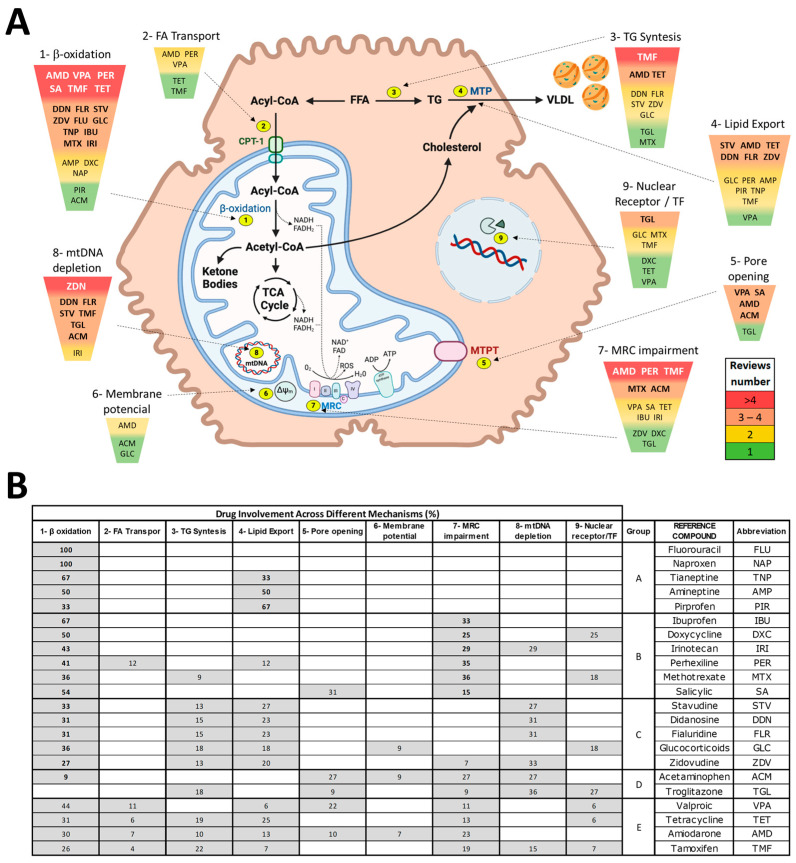
DIFLD mechanisms and associated drugs. (**A**) Different mechanisms have been identified or proposed to explain the onset of DIFLD by different drugs. They include: (1) impairment of FAO; (2) inhibition of fatty acid transport across the mitochondrial membranes; (3) increased de novo lipid synthesis; (4) reduction of lipid export by the inhibition of MTP; (5) induction of the MPT pore opening; (6) dissipation of the MTP; (7) impairment of MRC/OXPHOS (I–IV represent the respective complexes of the MRC, while C represents cytochrome c); (8) mtDNA damage and depletion; (9) NR/transcriptomic alterations (alteration of TFs/NRs by modifying their expression levels or by direct agonist/antagonist activity). The triangle and the colour gradient/font size represents the relative number of times the association between the drug and the mechanism has been reported in literature reviews. (**B**) Percentage of drug participation in the different steatogenic mechanisms, based on prevalence in reviewed literature. Most drugs are associated with more than one mechanism, yet some of them are more frequently reported. Drug groups: (A) drugs predominantly altering β-oxidation or lipid transport; (B) drugs predominantly involved in β-oxidation or MRC impairment; (C) drugs involved in β-oxidation, triglyceride synthesis, and lipid export primarily; (D) not involved in β-oxidation but implicated in various other mechanisms; and (E) drugs involved in β-oxidation, fatty acid transport, lipid export, and several other mechanisms. For more details, see Table S1.

The onset of DIFLD involves diverse molecular mechanisms and pathways, each contributing to lipid accumulation within hepatocytes (summarised in Figure 4).

Understanding the molecular mechanisms underlying drug-induced steatosis involves a complex interplay of various pathways. These mechanisms and pathways collectively contribute to the accumulation of lipids within hepatocytes when exposed to drugs causing steatosis (Figure 4).

Following putative mechanisms may be in play when identifying a drug’s mode of action:

### 6.1. Inhibition of Mitochondrial Fatty Acid β-Oxidation (FAO)

Once inside mitochondria, fatty acids undergo β-oxidation, a series of enzymatic reactions that result in the production of acetyl-CoA and, ultimately, ATP. A first mechanism by which drugs can cause steatosis is by directly inhibiting mitochondrial FAO or through a primary effect on the mitochondrial genome or the respiratory chain itself [44].

Some drugs impair FAO by interacting with different mitochondrial enzymes. Compounds such as ibuprofen, amiodarone, tamoxifen, or valproate (or their metabolites) inhibit FAO enzymes [22]. Paracetamol seems to inhibit FAO enzymes via the reactive metabolite N-acetyl-p-benzoquinone imine [45,46], which would explain why this compound causes DIFLD in some individuals [29].

### 6.2. Inhibition of Fatty Acid Transport across Mitochondrial Membranes

The transport of fatty acids across the mitochondrial membrane is a crucial process in cellular metabolism, particularly in the context of energy production through FAO. This transport involves several steps. Fatty acids are initially activated in the cytosol by attaching to coenzyme A (CoA), forming fatty acyl-CoA. Fatty acyl-CoA cannot directly cross the inner mitochondrial membrane. Instead, it forms a complex with carnitine, a process catalysed by the enzyme carnitine palmitoyl-transferase (CPT) I, the rate-limiting enzyme in mitochondrial FAO. The resulting fatty acylcarnitine is transported across the inner mitochondrial membrane. Once inside the mitochondrion, the fatty acylcarnitine is converted back to fatty acyl-CoA by CPT-II [13,47].

Inhibition of CPT-I can distort fatty acid transport across the mitochondrial membrane, leading to severe side effects such as FAO inhibition and hepatic steatosis [13,22,48]. Valproate restricts FAO by interacting with the acyl-CoA formation. CPT-I is among the key targets inhibited by valproate, amiodarone, and tamoxifen. Interestingly, troglitazone is able to inhibit long-chain acyl-CoA synthase, thus impairing its mitochondrial entry. Drugs can also sequester CoA and/or L-carnitine, which are essential cofactors, and impair effective mitochondrial FAO. This seems to be the case for valproate, salicylic acid, and ibuprofen, as well [49,50].

### 6.3. Increased De Novo Lipid Synthesis (DNL)

While mitochondrial dysfunction and compromised FAO have conventionally been recognized as pivotal factors in DIFLD [44], other researchers have pinpointed additional mechanisms that could also contribute to steatosis, even in the absence of severe mitochondrial dysfunction and with mild-to-moderate inhibition of mitochondrial FAO. Drugs like amiodarone trigger an increase in DNL, a phenomenon that potentially develops through several mechanisms including endoplasmic reticulum stress [51] and induction/activation of lipogenic transcription factors: LXR, PXR, PPARγ, SREBP1c and ChREBP. Elevated concentrations of insulin and glucose in the plasma stimulate the activation of SREBP-1c and ChREBP. These transcription factors, in turn, upregulate the hepatic expression of pivotal enzymes involved in glycolytic metabolism, such as glucokinase and l-pyruvate kinase, as well as enzymes facilitating DNL, including acetyl-CoA carboxylase and fatty acid synthase. Other transcription factors that could play a significant role in DNL are the NRs LXR, PPARγ and PXR, which can be activated by different endogenous and exogenous ligands [13].

### 6.4. Reduction in Lipid Export by the Inhibition of Microsomal Triglyceride Transfer Protein (MTP)

Impairment of VLDL secretion has also been shown as a functional mechanism in drug-induced steatosis [51]. MTP is involved in the transfer of triglycerides to ApoB and assembly of VLDL in the liver. Inhibition of MTP activity can lead to a reduction in lipidation of nascent VLDLs and, consequently, an increase in cytoplasmic lipid droplets. Previous research has shown that MTP inhibitors, while effective in lowering serum LDL, can cause dose-dependent hepatic steatosis and variable degree of transaminitis. Genetic studies have also identified an interaction between MTP and MASLD, suggesting a role for MTP in the development of hepatic steatosis [52,53]. Regarding causative drugs, there is evidence that amineptine, amiodarone, pirprofen, tetracycline and tianeptine directly inhibited MTP activity, decreased TG in the luminal VLDL fraction and decreased in vivo hepatic lipoprotein secretion [54].

### 6.5. Induction of Mitochondrial Permeability Transition (MPT) Pore Opening

An additional mechanism contributing to mitochondrial dysfunction involves the opening of MPT pores. This occurrence significantly hampers ATP synthesis by compromising the integrity of the inner mitochondrial membrane and the mitochondrial membrane potential that ensures ATP synthesis. As MPT pores open in multiple mitochondria, ATP stores deplete rapidly. Consequently, an abrupt increase in intracellular calcium levels follows, precipitating cell necrosis. This connection between ATP and calcium is attributed to the pivotal role of ATP in facilitating the activity of the plasma membrane calcium ATPase, responsible for pumping out calcium from the cell. The resultant outcome is cell death (necrosis) followed by inflammation. Valproate has been shown to induce MPT pore opening, contributing along with other mechanisms to mitochondrial dysfunction [50,55].

### 6.6. Dissipation of the Mitochondrial Transmembrane Potential (ΔΨm)

The ΔΨm generated by inner mitochondrial membrane proton pumps (Complexes I, III and IV) is an essential component in the process of energy storage (ATP) during oxidative phosphorylation. Together with the proton gradient, ΔΨm forms the transmembrane potential of hydrogen ions that is used to produce ATP. Drugs that have dissociable protons and permeate bilayers (e.g., lipophilic weak acid) can dissipate ΔΨm causing mitochondrial dysfunction; amiodarone is an example [35,50,56].

### 6.7. Impairment of the Mitochondrial Respiratory Chain (MRC)/Oxidative Phosphorylation (OXPHOS)

Severe inhibition of the MRC can lead to a secondary impairment of mitochondrial FAO, as these two metabolic pathways are closely interconnected. The MRC is essential for the continuous replenishment of FAD and NAD^+^, which are vital for the enzymatic activities of FAO enzymes, including acyl-CoA dehydrogenases and 3-hydroxyacyl-CoA dehydrogenases. Notably, inhibition of FAO following MRC impairment could manifest with drugs like amiodarone and tamoxifen [23,57,58]. These drugs can become positively charged in the mitochondrial intermembrane space. This allows them to enter the matrix due to the membrane potential Δψm. The buildup of these positively charged compounds in mitochondria inhibit MRC and uncouples OXPHOS, leading to the inhibition of FAO enzymes. While these drugs can directly inhibit FAO at low concentrations within mitochondria, higher concentrations are needed to affect the MRC [35,58,59,60]. MRC activity can also be reduced by tetracyclines [35,61]. However, it remains uncertain whether these medications hinder mitochondrial FAO by affecting the MRC or through a direct mechanism.

### 6.8. Mitochondrial DNA (mtDNA) Damage, Degradation, and Depletion

Drugs can directly damage mtDNA, resulting in diminished number of mitochondria and mitochondrial proteins [62]. Essential mitochondrial proteins are encoded by genes residing in both mtDNA and nuclear DNA. Mitochondrial proteins undergo a coordinated synthesis facilitated by the collaborative action of both nuclear and mitochondrial genetic machinery. Proteins synthesised within mitochondria from mtDNA undergo modification, folding and assembly without leaving the mitochondria. Subsequently, these proteins are transported to ensure accurate localization within the mitochondria. mtDNA depletion leads to a deficiency in crucial mitochondrial proteins, resulting in impaired MRC (mtDNA encodes 13 MRC polypeptides) and the subsequent inhibition of FAO [13,35]. This phenomenon is exemplified by antiviral drugs such as didanosine, fialuridine, stavudine, and zidovudine, which inhibit mtDNA polymerase γ [23,35,62,63], leading to reduced mtDNA and gene expression, impeding the tricarboxylic acid cycle and correlating with lactic acidosis [23,64,65]. Furthermore, tamoxifen and tacrine, both interacting with mitochondrial topoisomerases, have also been observed to induce hepatic mtDNA depletion, although the extent to which this mechanism contributes to pathophysiology remains uncertain [57,59,66].

Damage to mtDNA can be instigated by the generation of ROS, reactive nitrogen species, and/or reactive metabolites induced by drugs. For example, substances such as APAP and troglitazone can cause mtDNA strand breaks, ultimately resulting in a decrease in mtDNA levels [67,68]. Indeed, mtDNA molecules with extensive strand breaks due to damage can undergo swift degradation facilitated by mitochondrial endonucleases [69,70,71]. NRTIs can also lead to the accumulation of the oxidised base 8-hydroxydeoxyguanosine (8-OH-dG) in the mtDNA of liver and muscle. This accumulation may have implications for mtDNA base modification, as evidenced by the detection of mtDNA point mutations in some patients undergoing NRTI treatment. These mutations may originate from two potential sources: first, the misinterpretation of 8-OH-dG by DNA polymerase γ during mtDNA replication; and second, the diminished repair capacity of polymerase γ induced by NRTIs [62,72,73]. Consequently, certain drugs have the potential to induce both quantitative and qualitative alterations in mtDNA through their interaction with mitochondrial enzymes involved in mtDNA replication and maintenance.

### 6.9. Nuclear Receptor/Transcriptomic Alterations

Several pioneering studies have investigated the effect of well-characterised steatotic drugs on mouse liver gene expression profiles. Surprisingly, these studies revealed that drugs inducing steatosis could also induce significant changes in the liver transcriptome. Microarray analysis uncovered a substantial number of genes responsive to such drugs: 96 genes with tetracycline [74], 414 genes with tamoxifen [75], 908 genes with methotrexate [76], and 1910/1325 genes for acute/chronic valproate exposure [77,78]. The precise molecular mechanism responsible for these significant changes in mRNA expression remains incompletely understood. These alterations could potentially stem from direct toxic exposure or manifest as downstream consequences. Nevertheless, factors such as the inhibition or activation of TFs, notably NRs, as well as changes in epigenetic markers and microRNAs, may contribute to the considerable transcriptomic impact of steatogenic drugs.

Similar results have been found in human hepatic cells exposed to drugs causing steatosis. We found evidence suggesting that drugs such as tetracycline, valproate, doxycycline, and amiodarone cause severe alterations in the expression of 47 TFs and coregulators related to energy metabolism and liver phenotype, among them FOXA1, HEX, and SREBP1c, all of them involved in liver lipid metabolism [79,80].

Different chemicals can trigger oxidative, genotoxic, and proteotoxic stresses, which induce cellular responses devoted to restoring homoeostasis. The most important defensive responses involve TFs such as Nrf2 (antioxidant response) and Xbp1 (unfolded protein response), both related to lipid metabolism pathways as well. Other pathways including the immunomodulatory transcription factors NF-κB and STAT are also implicated in inflammatory responses to xenobiotic exposure. Additionally, more specific mechanisms exist where xenobiotics can act as nuclear factor ligands, including the aryl hydrocarbon receptor, and the NR family of transcription factors [81].

Numerous NRs are implicated in regulating energy homeostasis and biotransformation, forming a complex network that interconnects fatty acids, cholesterol, and xenobiotic metabolism. Consequently, multiple NRs and their ligands are speculated to exert a substantial influence on liver fat metabolism and accumulation. Our investigation delved into the steatogenic potential of 76 distinct NR ligands in human hepatocytes and hepatoma cells overloaded with fatty acids. Our findings reveal that 18% of the scrutinised NR ligands exacerbate steatosis. Notably, ligands targeting PPARγ (such as thiazolidinediones), LXR (e.g., paxilline and 24(S),25-epoxycholesterol), PXR (hyperforin), CAR (3alpha,5alpha-androstenol), ERα (tamoxifen), FXR (Z-guggulsterone), VDR (25-hydroxyvitamin D3), as well as certain retinoids and farnesoids, exhibited a significant steatogenic effect. Through a comparative analysis of the steatogenic effect of NR ligands and NR expression levels, we deduce that ligands for LXR, PXR, RAR, and PPARγ likely induce fat accumulation through a mechanism reliant on NRs [82].

In a recent study, it was shown that the majority of steatogenic drugs, including valproate, doxycycline, tetracycline, cyclosporine A, and tianeptine, had a negative impact on the expression of the atypical NR small heterodimer partner (SHP). However, tamoxifen, amiodarone, and zidovudine did not repress SHP. Investigation into the molecular mechanism showed that steatotic drugs trigger stress signals that target C/EBPa, which consequently repress SHP [83].

Stimulation of hepatic steatosis by drugs such as interferon-α, glucocorticoids, tamoxifen, troglitazone, and nifedipine could be triggered by activation of transcription factors that induce lipid synthesis (LXR, PXR, PPARγ, SREBP1c and ChREBP). Moreover, glucocorticoid receptor (GR) activation plays a central role in glucocorticoid-induced hepatic lipogenesis, and steatosis. Alternatively, mitochondrial, peroxisomal, and microsomal FAO is strongly regulated by PPARα, which can be stimulated by endogenous fatty acids or synthetic drugs (fibrates), but also antagonized or inhibited by other drugs [13]. This repression of PPARα by drugs would lead to lipid accumulation and steatosis.

Based on mechanistic considerations in drug-induced steatosis, [84] identified 19 genes exhibiting dose-dependent responses and 10 genes showing time-dependent patterns. Notably, this study delineated 9 genes (ANGPTL4, FABP7, FADS1, FGF21, GOT1, LDLR, GK, STAT3 and PKLR) as signature markers for predicting drug-induced steatosis. Additionally, cross-tabulation analysis revealed that 9 genes were consistently regulated in ≥10 instances across various conditions, encompassing genes involved in glucose metabolism, lipid transport, lipogenesis, and signalling pathways. Furthermore, a comparison between drugs inducing phospholipidosis and/or steatosis uncovered 26 commonly regulated genes, including from the signature markers (PKLR, GK, FABP7 and FADS1).

Other alternative mechanisms include drug-induced epigenetic alterations such as miRNome and methylome deregulation. Valproate for instance modulated the expression and DNA methylation level of NRs and their target genes involved in the adverse outcome pathway of steatosis thereby inhibiting FAO and increasing uptake of fatty acid into the hepatocytes [85].

The nucleus serves as the primary site for most MIEs cited in current AOPs of liver steatosis. However, scientific literature increasingly indicates that mitochondria and their functional impairment, induced by several mechanisms, take centre stage. This impairment can be directly induced by various drugs [50,86].

## 7. Clustering of Steatotic Drugs by Mechanism of Action and Clinical Outcome

**Figure 5:**
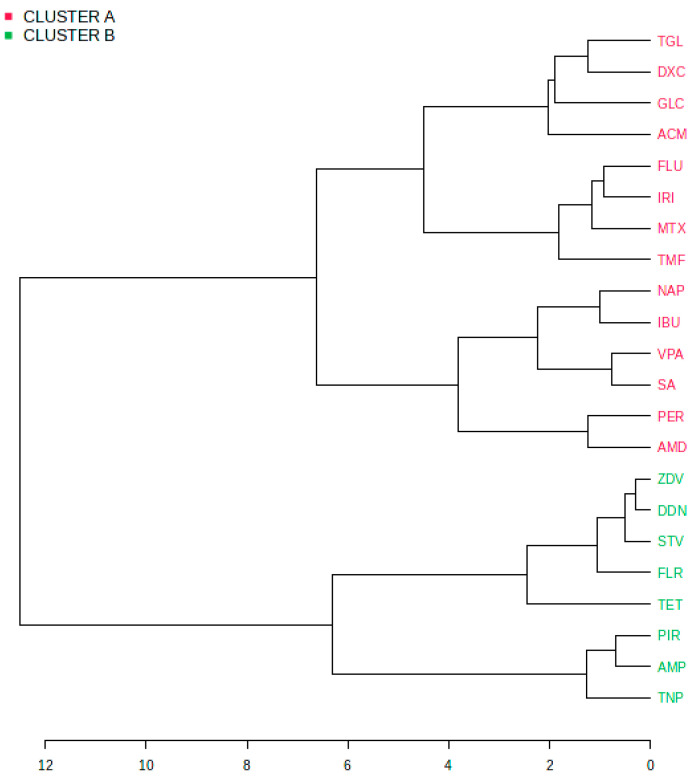
Hierarchical dendrogram of DIFLD drugs. Input data for each drug included the number of reports in literature reviews associated with a given mechanism of toxicity and the DIFLD outcomes as shown in Supplementary Table S1. The dendrogram was generated by MetaboAnalyst, where the distance measured by Pearson is shown. Two major clusters became evident. Cluster A included several prototypical steatotic drugs that most frequently cause MRC/OXPHOS impairment and steatohepatitis. Cluster B drugs were all associated with an impaired lipid export. ACM (Acetaminophen), AMD (Amiodarone), AMP (Amineptine), DDN (Didanosine), DXC (Doxycycline), FLR (Fialuridine), FLU (Fluorouracil), GLC (Glucocorticoids), IBU (Ibuprofen), IRI (Irinotecan), MTX (Methotrexate), NAP (Naproxen), PER (Perhexiline), PIR (Pirprofen), SA (Salicylic), STV (Stavudine), TET (Tetracycline), TGL (Troglitazone), TMF (Tamoxifen), TNP (Tianeptine), VPA (Valproic) and ZDV (Zidovudine).

Through an extensive review of the literature, we precisely collected clinical and mechanistic information of the drugs most commonly implicated in DIFLD (Supplementary Table S1). This enabled us to semi-quantitatively assess their participation in various DIFLD outcomes (macro- and microsteatosis, steatohepatitis), as well the involvement of the different potential toxicity mechanisms. Leveraging this information, we formulated a hierarchical dendrogram to explore the clustering patterns of drugs (Figure 5).

Two distinct clusters were identified. Compounds belonging to the same therapeutic family tend to group together. Thus, for example, in the larger cluster A (in red), the antiarrhythmics amiodarone and perhexiline were grouped in a subcluster, and the chemotherapeutics fluorouracil, irinotecan, methotrexate, and tamoxifen were grouped in a different subcluster evidencing the similarities in the mechanism of liver damage and final clinical outcome. Additionally, most antibiotics and NSAIDs fell within the cluster A, which also included several prototypical steatotic drugs such as valproate, amiodarone, tamoxifen, and methotrexate, along with other chemotherapeutics and antiarrhythmics. The smaller cluster B (in blue) included NRTIs (zidovudine, didanosine, stavudine, and fialuridine) grouped in the same subcluster, antidepressants (tianeptine and amineptine), tetracycline and pirprofen.

Interestingly, drugs expected to promote/aggravate steatosis in patients with pre-existing MASLD or associated diseases (GROUP 1 in Table 2) are all found in cluster A, while those in GROUP 3, showing a great tendency to transit to steatohepatitis and further to fibrosis, belong to cluster B.

Another interesting observation is that drugs in cluster B were associated with an impaired lipid export, whereas those in cluster A most frequently caused MRC/OXPHOS impairment and steatohepatitis (Figure 5).

## 8. Consistency of Identified DIFLD Mechanisms with Current Adverse Outcome Pathways (AOPs)

AOPs are novel roadmaps in toxicology and human risk assessment to more accurately describe the nature and consequences of the toxic phenomenon. They aim to provide a clear-cut mechanistic representation of key toxicological events in different layers of biological organisation. AOPs share a common structure consisting of a MIE, a series of intermediate steps and key events, and an adverse outcome. AOPs provide a framework that links the MIE to an adverse outcome through structured toxicological knowledge.

Several AOPs have been proposed to describe drug-induced liver steatosis in AOP-Wiki (https://aopwiki.org/, accessed on 7 March 2024). The nucleus serves as the primary site for most MIEs cited in current AOPs of liver steatosis. All of them give major relevance to NR/TF activation as the MIE. Many NR ligands have been linked specifically to steatosis, including LXR, PPARα, PPARγ, PXR, GR, FXR, CAR, ER, RAR, and AhR, yet AhR does not belong to the NR family.

The first AOP for steatosis was proposed within the framework of a European Community research initiative to address ‘Safety Evaluation Ultimately Replacing Animal Testing’ (SEURAT). This initiative embraced a toxicological mode-of-action framework to elucidate how various substances could potentially compromise human health. Steatosis was selected as a proof-of-concept AOP, and NR binding was chosen as the MIE. Among the several NR, LXR was chosen since the LXR agonist T0901317 was proposed as a reference chemical for liver steatosis [87].

This first AOP was taken as a starting point, and other NRs were incorporated as MIEs as well [88,89,90,91,92,93,94]. Presently, most, if not all AOPs for steatosis in AOP-Wiki identify a NR or another TF as the MIE. This observation prompts consideration that chemical-induced steatosis typically arises from an intricate interplay between a chemical and a NR/TF, implying that this interaction represents the primary and pivotal MIE. All the other postulated mechanisms would be intermediate key events that arise subsequently. Therefore, it is imperative to highlight that other equally crucial factors listed above, which directly influence lipid homeostasis in drug-induced steatosis, are not accounted for in the current AOPs.

Upon conducting this exhaustive literature review, it became evident that the current AOPs fall short in encapsulating the intricate biological mechanisms involved in chemically induced liver steatosis. This observation echoes the findings of [95], who already identified discrepancies in gene regulation within the steatosis AOP when studying cyproconazole. A global down-regulation of CYP, SULT, and UGT genes was observed, which might look surprising considering that activation of PXR, CAR, or AhR would result in their upregulation. It is noteworthy that processes like inflammation can inhibit NR pathways. Up-regulation of JUN (part of AP1) mediates the release of cytokines such as IL-8. Moreover, there is evidence of interconnection with NFκB. Hence, an initial hypothesis suggests that inflammation-related mechanisms could be activated simultaneously with triglyceride accumulation, potentially counteracting any induction by activated NRs.

Another interesting observation drawn from the mining of public toxicogenomics databases suggests that mitochondrial toxicity rather than NR activation is likely an underestimated MIE in chemical-induced steatosis. Considering mitochondrial toxicity as a pivotal MIE for steatosis aligns with the established toxicological impacts of extensively researched steatosis-inducing substances like amiodarone, valproic acid, and tetracycline, along with various other pharmaceutical and agricultural chemicals [96].

Many studies support the involvement of NRs in steatosis, but it is also true that others show that such NR interactions alone do not necessarily lead to steatosis. For instance, a clinical study found that the thiazolidinedione pioglitazone, a PPARγ agonist, significantly reduced steatosis contrary to what we would expect if PPARγ agonism is a steatosis MIE. Troglitazone also alleviated steatosis in MASLD patients [97]. In the same direction, another clinical trial found that a FXR agonist reduces steatosis. FXR activation was reported to decrease SREBP1C gene and increase PPARα gene leading to decrease in lipid synthesis and increase in FAO. Multiple animal studies also report that FXR agonism has an anti-steatotic effect. CAR activation, another MIE in the previously proposed AOPs, was reported to repress the target genes of LXR, a key gene whose activation is known to up-regulate lipid synthesis [98].

Another intriguing example is that of tamoxifen, which selectively binds to ER and exhibits both estrogenic agonist and antagonist effects in different parts of the body [99,100,101]. Tamoxifen has been implicated in the exacerbation of steatohepatitis/steatosis, especially in obese individuals, through its direct effect on mitochondrial respiration and lipid metabolism interference. In contrast to this, the drug has been found to improve hepatic steatosis and glucose intolerance in mice via the inhibition of JNK/MAPK signaling [102,103]. Thus, tamoxifen’s effect on hepatic steatosis is believed to be independent of its action on ER. 

Finally, pro- and anti-steatogenic roles are simultaneously suggested for the AhR (e.g.,) [27]. Some studies examining the role of AhR in fatty acid metabolism and MASLD reported that TCDD-induced AhR activation resulted in hepatic steatosis and further inflammation and fibrosis. Conversely, treatment with endogenous AhR agonists, (e.g., cinnabarinic acid, indole, and indole-3 acetic acid), attenuated steatosis [104].

**Figure 6:**
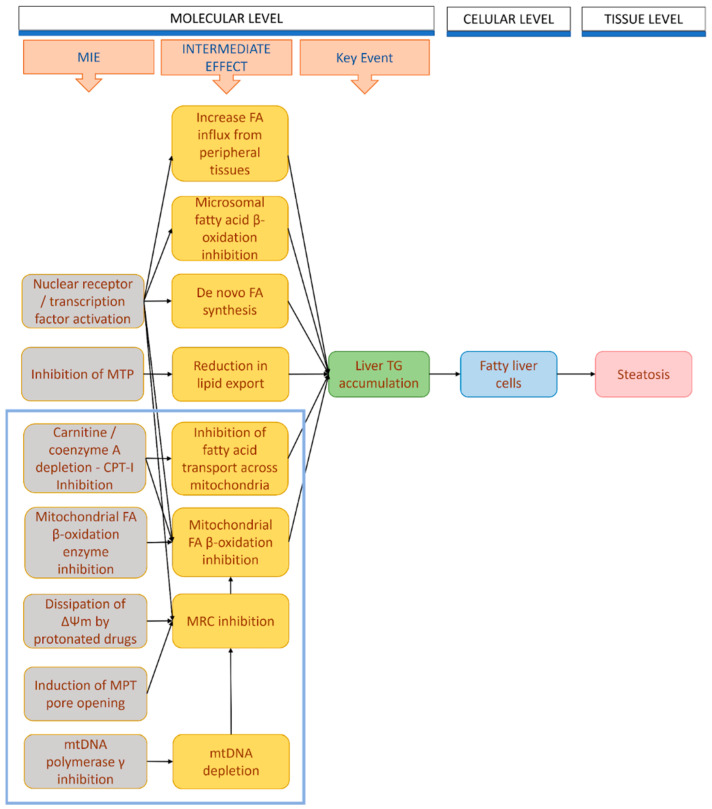
An integrative perspective on acknowledged mechanisms, as well as other potential MIEs influencing liver steatosis AOPs. The blue box serves to gather MIEs and intermediate effects occurring exclusively within the mitochondria, consequently impacting mitochondrial functionalities. FA (fatty acid), TG (triglyceride).

These reports underscore the contentious role of NRs in steatosis. Consequently, without additional experimental inquiries elucidating the interplay between NR activation and steatosis, it may be premature to definitively designate specific NRs (such as FXR) as the primary and almost unique MIEs, as it appears in the current AOPs of steatosis. This perspective only captures a fraction of the phenomenon’s complexity. The scientific literature increasingly indicates that mitochondria and their functional impairment, take centre stage. Integrative efforts are mandatory to assimilate novel MIEs, as envisaged in the list of other acting mechanisms, as we propose in Figure 6, demanding rigorous weight-of-evidence evaluations prior implementation into drug-induced steatosis AOP.

## Figures and Tables

**Table 1:** Representative clinical manifestations observed in DIFLD caused by different drugs. Data extracted from LiverTox.

Anamnesis	Biochemical Alterations	Additional Histopathological Findings	Exploratory Manifestation	Reference Compound
Minimal initial exploratory signs and symptoms	Aminotransferases, ALP (mild/moderate)	ND	Minimal	Irinotecan
	Liver test (normal/minimal and transient until cirrhosis)	Portal hypertension, cirrhosis, fibrosis	Absent until cirrhosis	Methotrexate
	ALT	Hepatitis, inflammation, fibrosis, ballooning degeneration, Mallory bodies	Absent until fibrosis	Tamoxifen
	Aminotransferase	Portal inflammation	Minimal	5-Fluorouracil
Mainly jaundice	Liver test (variable)	Ballooning, inflammation, fibrosis, Mallory bodies, abnormal mitochondria and phospholipid laden lysosomes	Jaundice	Amiodarone
	ALT (variable)	Inflammation	Jaundice	Valproic
Diverse exploratory signs and symptoms	Liver test (mild/moderate), lactic acidosis	Minimal inflammation and no obvious hepatocellular necrosis	Weakness, fever, fatigue, nausea and abdominal pain	Tetracycline
	ALT, ALP (minimal), lactic acidosis	Cholestasis, fibrosis, Mallory bodies	Fatigue, weight loss	Zidovudine
	Liver test (mild/moderate), lactic acidosis	Cholestasis, ballooning cell degeneration, fibrosis, Mallory bodies	Jaundice, nausea, vomiting, abdominal pain,	Stavudine
	ALT (moderate), lactic acidosis	Cholestasis, fibrosis	Nausea, vomiting, abdominal pain	Didanosine

**Table 2:** Classification of DIFLD drugs according to clinical status and pathological features. Based on literature review.

Drug Group	Clinical Features	Previous Status of the Patient
**GROUP 1** (acetaminophen, ibuprofen, naproxen, irinotecan, methotrexate, tamoxifen)	Weak hepatotoxic drugs, only causing steatosis in MASLD patients	Promote/aggravate pre-existing steatosis, MASLD, obesity, metabolic syndrome, cardiometabolic risk factors
**GROUP 2** (amiodarone, valproic acid, tetracycline)	Usually causing macro- or microvesicular steatosis and occasionally steatohepatitis and fibrosis	Not relevant
**GROUP 3** (zidovudine, stavudine, didanosine)	Rapid progress towards steatohepatitis, fibrosis, cholestasis, lactic acidosis	Not relevant

**Table 3:** Relative incidence of key histological features and clinical outcomes of selected DIFLD drugs.

Therapeutic Group	Compound	SH	Micro	Macro	Fibrosis (LiverTox)
Anticonvulsants	Valproic	1	9	1	
Antidiabetics	Troglitazone	0	1	0	
Anti-inflammatories	Glucocorticoids	0	5	5	
Antibiotics	Doxycycline	0	0	0	
	Tetracycline	0	8	0	
Antidepressant	Tianeptine	0	0	0	
	Amineptine	0	0	1	
Antiarrhythmics	Amiodarone	6	5	8	*
	Perhexiline	3	1	0	
Chemotherapeutic	Fluorouracil	4	2	7	
	Irinotecan	8	1	4	
	Methotrexate	7	2	9	*
	Tamoxifen	8	1	9	*
NRTIs	Didanosine	0	7	2	*
	Fialuridine	0	3	0	
	Stavudine	0	7	2	*
	Zidovudine	0	7	2	*
NSAID	Acetaminophen	0	0	1	
	Ibuprofen	0	7	2	
	Naproxen	0	4	0	
	Pirprofen	0	1	0	
	Salicylic	0	5	1	

Numbers indicate the number of articles reporting the specific histological features. Micro: microsteatosis; Macro: macrosteatosis; SH: steatohepatitis. Asterisks denote fibrosis reported in LiverTox.

## Supplementary Materials

The following supporting information can be downloaded at: https://www.mdpi.com/article/10.3390/ijms25105203/s1. References [10,13,15,16,26,27,42,49,50,56,86,105,106,107,108] are cited in the Supplementary Materials.

## Abbreviations

ACM	Acetaminophen
ALP	Alkaline phosphatase
ALT	Alanine aminotransferase
AMD	Amiodarone
AMP	Amineptine
AOPs	Adverse outcome pathways
CoA	Coenzyme A
CPT	Carnitine palmitoyl-transferase
DDN	Didanosine
DIFLD	Drug-induced fatty liver disease
DISH	Drug-induced steatohepatitis
DNL	De novo lipogenesis
DXC	Doxycycline
FA	Fatty acid
FAO	Fatty acid β-oxidation
FLR	Fialuridine
FLU	Fluorouracil
GLC	Glucocorticoids
IBU	Ibuprofen
IRI	Irinotecan
MASH	Metabolic dysfunction-associated steatohepatitis
MASLD	Metabolic dysfunction-associated steatotic liver disease
MetALD	Metabolic alcohol-related liver disease
MPT	Mitochondrial permeability transition
MRC	Mitochondrial respiratory chain
mtDNA	Mitochondrial DNA
MTP	Microsomal triglyceride transfer protein
MTX	Methotrexate
NAP	Naproxen
NR	Nuclear receptor
NRTIs	Nucleoside reverse transcriptase inhibitors
NSAIDs	Nonsteroidal anti-inflammatory drugs
OXPHOS	Oxidative phosphorylation
PER	Perhexiline
PIR	Pirprofen
ROS	Reactive oxygen species
SA	Salicylic
SLD	Steatotic liver disease
STV	Stavudine
TET	Tetracycline
TF	Transcription factors
TG	Triglyceride
TGL	Troglitazone
TMF	Tamoxifen
TNP	Tianeptine
VLDL	Very low-density lipoprotein
VPA	Valproic
ZDV	Zidovudine
ΔΨm	Mitochondrial transmembrane potential
