# Supplementary material for: Drug-Induced Fatty Liver Disease (DIFLD): A Comprehensive Analysis of Clinical, Biochemical, and Histopathological Data for Mechanisms Identification and Consistency with Current Adverse Outcome Pathways
Source: Int J Mol Sci. 2024 May 10;25(10):5203. doi: 10.3390/ijms25105203 (PMC11121209; doi:10.3390/ijms25105203)
Supplement: Supplementary file 1 [file ijms-25-05203-s001.zip › ijms-2956953-supplementary.pdf]

Terapeutic Group	COMPOUND	β oxidation 1	FA Transpor 2	TG Syntesis 3	Lipid Export 4	Pore opening 5	ΔΨm 6	MRC/Ox Phos 7	mtDNA 8	Nuclear 9	Abr.	SH	m	M
Anticonvulsant	Valproic	1| 4| 6| 7 10| 12| 13| 14	10| 13		10	1| 6| 7| 10		4| 7		13	VPA	9	1|2|3|4|7 8|9|12|15	1
Antidiabetic	Troglitazone			6| 7		6		6	6| 7| 10| 12	6| 7| 12	TGL		6	
Anti-inflammatories	Glucocorticoids	4| 7| 16| 12		7| 12	4| 16		7			7| 12	GLC		2|3|8|9 12	3|4|7|9 12
Antibiotic	Doxycycline	10| 14						6		13	DXC			
Antibiotic	Tetracycline	7| 10| 12| 13 14	13	10| 12| 13	7| 10| 12| 13			6| 12		13	TET		2|3|4|7 8|9|12|15	
Antidepressant	Tianeptine	6| 7| 10| 14			10| 13						TNP			
	Amineptine	7| 14			10| 13						AMP			1
Antiarrhythmic	Amiodarone	1| 4| 6| 7| 10 12| 13| 14| 16	10| 13	3| 7| 12	7| 10| 12| 13	1| 6| 10	10| 16	1| 6| 7| 10 12| 13| 16			AMD	1|3|5|7 9|12	1|2|8|12 15	1|2|3|4 7|8|9|12
	Perhexiline	1| 6| 12| 13 14| 16	10| 13		7| 16			1| 6| 10| 12 13| 16			PER	4|5|15	15	
Chemotherapy	Fluorouracil	1| 3| 7| 12									FLU	1|3|9|15	2|8	1|3|4|9 11|12|15
	Irinotecan	1| 3| 7						1| 13	1| 13		IRI	1|3|4|6 7|9|13|15	15	1|3|9|11
	Methotrexate	1| 7| 10| 12		10				1| 4| 7| 12		7| 13	MTX	3|4|6|7 9|12|13	2|8	2|3|4|7|8 9|11|12|15
	Tamoxifen	1| 4| 6| 7 10| 12| 13	13	3| 4| 7| 10 12| 13	7| 12			1| 6| 10| 12 13	1| 10| 12| 13	12| 13	TMF	3|4|5|6 7|9|12|13	9	2|3|4|7|8 9|11|12|15
NRTIs	Didanosine	3| 6| 7| 12		3| 12	3| 7| 12				4| 6| 7| 10		DDN		2|3|7|8 9|12|16	2|8
	Fialuridine	3| 6| 7| 12		3| 12	3| 7| 12				1| 7| 6| 10		FLR		7|9|16	
	Stavudine	3| 6| 7| 10 12		3| 12	3| 7| 10| 12				1| 6| 7| 10		STV		2|3|7|8 9|12|16	2|8
	Zidovudine	3| 6| 7| 12		3| 12	3| 7| 12			10	1| 4| 6| 7 10		ZDV		2|3|8|9 12|15|16	2|8
NSAID	Acetaminophen	10				1| 6| 14	14	1| 6| 14	1| 7| 8		ACM			1
	Ibuprofen	4| 6| 7| 10						1| 6			IBU		1|3|4|7 8|9|16	9|15
	Naproxen	4| 7									NAP		3|4|7|8	
	Pirprofen	13			10| 13						PIR		3	
	Salicylic	1| 4| 6| 7 10| 12| 14				1| 4| 6| 7		1| 6			SA		1|3|4|7|9	9

ΔΨm = Potencial de membrana mitocondrial; SH= Steatohepatitis; m = Microvesicular steatosis; M = Macrovesicular steatosis. **1**. Impairment of β-oxidation **2**. Inhibition of fatty acid transport across the mitochondrial membranes; **3**. Increased de novo lipid synthesis; **4.** Reduction in lipid export by the inhibition of microsomal triglyceride transfer protein (MTP) activity; **5.** Induction of mitochondrial permeability transition (MPT) pore opening; **6.** Dissipation of the mitochondrial transmembrane potential; **7.** Impairment of mitochondrial respiratory chain (MRC) / oxidative phosphorylation (OXPHOS); **8**. Mitochondrial DNA

Table S1. Prevalence in literature reviews associated with the involvement of the different mechanisms of toxicity and the DIFLD outcomes of each drug. Detailed references for the main steatotic drugs referenced in literature reviews analysed for their role in causing or exacerbating steatosis, with respect to the hepatotoxicity mechanism, histopathological findings including microsteatosis (Micro) or macrosteatosis (Macro), and the clinical outcome Steatohepatitis (SH) to which they refer [1–16].

1. Stravitz, R.T.; Sanyal, A.J. Drug-Induced Steatohepatitis. *Clinics in Liver Disease* **2003**, *7*, 435–451, doi:10.1016/S1089-3261(03)00027-8.
2. Satapathy, S.K.; Kuwajima, V.; Nadelson, J.; Atiq, O.; Sanyal, A.J. Drug-Induced Fatty Liver Disease: An Overview of Pathogenesis and Management. *Annals of Hepatology* **2015**, *14*, 789–806, doi:10.5604/16652681.1171749.
3. Stine, J.G.; Chalasani, N. Chronic Liver Injury Induced by Drugs: A Systematic Review. *Liver International* **2015**, *35*, 2343–2353, doi:10.1111/liv.12958.
4. Rabinowich, L.; Shibolet, O. Drug Induced Steatohepatitis: An Uncommon Culprit of a Common Disease. *BioMed Research International* **2015**, *2015*, 1–14, doi:10.1155/2015/168905.
5. Patel, V.; Sanyal, A.J. Drug-Induced Steatohepatitis. *Clinics in Liver Disease* **2013**, *17*, 533–546, doi:10.1016/j.cld.2013.07.012.
6. Farrell, G. Drugs and Steatohepatitis. *Semin Liver Dis* **2002**, *22*, 185–194, doi:10.1055/s-2002-30106.
7. Begriche, K.; Massart, J.; Robin, M.-A.; Borgne-Sanchez, A.; Fromenty, B. Drug-Induced Toxicity on Mitochondria and Lipid Metabolism: Mechanistic Diversity and Deleterious Consequences for the Liver. *Journal of Hepatology* **2011**, *54*, 773–794, doi:10.1016/j.jhep.2010.11.006.
8. Bessone, F.; Dirchwolf, M.; Rodil, M.A.; Razori, M.V.; Roma, M.G. Review Article: Drug-induced Liver Injury in the Context of Nonalcoholic Fatty Liver Disease – a Physiopathological and Clinical Integrated View. *Aliment Pharmacol Ther* **2018**, *48*, 892–913, doi:10.1111/apt.14952.
9. Ortega-Alonso, A.; Andrade, R.J. Chronic Liver Injury Induced by Drugs and Toxins. *J of Digest Diseases* **2018**, *19*, 514–521, doi:10.1111/1751-2980.12612.
10. Gervasoni, C.; Cattaneo, D.; Filice, C.; Galli, M. Drug-Induced Liver Steatosis in Patients with HIV Infection. *Pharmacological Research* **2019**, *145*, 104267, doi:10.1016/j.phrs.2019.104267.
11. Teresa Donato, M.; Jose Gomez-Lechon, M. Drug-Induced Liver Steatosis and Phospholipidosis: Cell-Based Assays for Early Screening of Drug Candidates. *CDM* **2012**, *13*, 1160–1173, doi:10.2174/138920012802850001.
12. Pavlik, L.; Regev, A.; Ardayfio, P.A.; Chalasani, N.P. Drug-Induced Steatosis and Steatohepatitis: The Search for Novel Serum Biomarkers Among Potential Biomarkers for Non-Alcoholic Fatty Liver Disease and Non-Alcoholic Steatohepatitis. *Drug Saf* **2019**, *42*, 701–711, doi:10.1007/s40264-018-00790-2.
13. Dash, A.; Figler, R.A.; Sanyal, A.J.; Wamhoff, B.R. Drug-Induced Steatohepatitis. *Expert Opinion on Drug Metabolism & Toxicology* **2017**, *13*, 193–204, doi:10.1080/17425255.2017.1246534.
14. Schumacher, J.D.; Guo, G.L. Mechanistic Review of Drug-Induced Steatohepatitis. *Toxicology and Applied Pharmacology* **2015**, *289*, 40–47, doi:10.1016/j.taap.2015.08.022.
15. Serviddio, G.; Bellanti, F.; Sastre, J.; Vendemiale, G.; Altomare, E. Targeting Mitochondria: A New Promising Approach for the Treatment of Liver Diseases.
16. Ramachandran, R.; Kakar, S. Histological Patterns in Drug-Induced Liver Disease. *Journal of Clinical Pathology* **2009**, *62*, 481–492, doi:10.1136/jcp.2008.058248.
